# Endogenous and exogenous serotonin, but not sumatriptan, ameliorate seizures and neuroinflammation in the pentylenetetrazole-induced seizure model in rats

**DOI:** 10.1590/0004-282X-ANP-2021-0101

**Published:** 2022-01-31

**Authors:** Ibrahim Ethem Torun, Yasemin Baranoglu Kılınc, Erkan Kilinc

**Affiliations:** 1 Bolu Abant Izzet Baysal University, Faculty of Medicine, Department of Physiology, Bolu, Turkey. Bolu Abant Izzet Baysal University Faculty of Medicine Department of Physiology Bolu Turkey; 2 Bolu Abant Izzet Baysal University, Faculty of Medicine, Department of Pediatrics, Bolu, Turkey. Bolu Abant Izzet Baysal University Faculty of Medicine Department of Pediatrics Bolu Turkey

**Keywords:** Serotonin 5-HT1 Receptor Agonists, Fluoxetine, Seizures, Epilepsy, Inflammation, Agonistas do Receptor 5-HT1 de Serotonina, Fluoxetina, Convulsões, Epilepsia, Inflamação

## Abstract

**Background:**

Epilepsy has neuropsychiatric comorbidities such as depression, bipolar disorder, and anxiety. Drugs that target epilepsy may also be useful for its neuropsychiatric comorbidities.

**Objective:**

To investigate the effects of serotonergic modulation on pro-inflammatory cytokines and the seizures in pentylenetetrazole (PTZ)-induced seizure model in rats.

**Methods:**

Male Wistar rats were injected intraperitoneally with serotonin, selective serotonin reuptake inhibitor fluoxetine, 5-HT1B/D receptor agonist sumatriptan, or saline 30 min prior to PTZ treatment. Behavioral seizures were assessed by the Racine's scale. Concentrations of IL-1β, IL-6, and TNF-α in serum and brain tissue were determined by ELISA.

**Results:**

Serotonin and fluoxetine, but not sumatriptan, alleviated PTZ-induced seizures by prolonging onset times of myoclonic-jerk and generalized tonic-clonic seizures. The anti-seizure effect of fluoxetine was greater than that of serotonin. Likewise, serotonin and fluoxetine, but not sumatriptan, reduced PTZ-induced increases in the levels of IL-1β and IL-6 in both serum and brain tissue. None of the administered drugs including PTZ affected TNF-α concentrations.

**Conclusions:**

Our findings suggest that endogenous and exogenous serotonin exhibits anticonvulsant effects by suppressing the neuroinflammation. It seems that 5-HT1B/D receptors do not mediate anticonvulsant and anti-neuroinflammatory effects of serotonin.

## RESUMO

**Antecedentes:**

A epilepsia apresenta comorbidades neuropsiquiátricas como depressão, transtorno bipolar e ansiedade. Os medicamentos que visam o tratamento da epilepsia podem ser úteis para a epilepsia e suas comorbidades neuropsiquiátricas.

**Objetivo:**

Investigar os efeitos da modulação serotonérgica em citocinas pró-inflamatórias e as convulsões no modelo de convulsão induzida por pentilenotetrazol (PTZ) em ratos.

**Métodos:**

Ratos Wistar machos foram injetados intraperitonealmente com serotonina, inibidor seletivo da recaptação da serotonina fluoxetina, sumatriptano agonista do receptor 5-HT1B / D ou solução salina 30 min antes do tratamento com PTZ. As crises comportamentais foram avaliadas pela escala de Racine. As concentrações de IL-1β, IL-6 e TNF-α no soro e tecido cerebral foram determinadas por ELISA.

**Resultados:**

A serotonina e a fluoxetina, mas não o sumatriptano, aliviaram as convulsões induzidas por PTZ ao prolongar os tempos de início das convulsões mioclônicas e tônico-clônicas generalizadas. O efeito anticonvulsivo da fluoxetina foi maior do que o da serotonina. Da mesma forma, a serotonina e a fluoxetina, mas não o sumatriptano, reduziram os aumentos induzidos por PTZ nos níveis de IL-1β e IL-6 no soro e no tecido cerebral. Nenhum dos medicamentos administrados, incluindo PTZ, alterou as concentrações de TNF-α.

**Conclusões:**

Nossos achados sugerem que a serotonina endógena e exógena exibe efeitos anticonvulsivantes por suprimir a neuroinflamação. Aparentemente, os receptores 5-HT1B / D não medeiam os efeitos anticonvulsivantes e anti-neuroinflamatórios da serotonina.

## INTRODUCTION

Epilepsy is one of the most common neurological disorders and it is characterized by recurrent seizures. It affects over 70 million people worldwide^1^. Although it is widely accepted that an impairment between excitatory and inhibitory neurotransmission leads to epileptic seizures, the exact mechanisms underlying this imbalance are still unclear. However, increasing evidence suggests that neuroinflammation, as a trigger for the seizures, is implicated in the pathophysiology of epilepsy^2^^-^^4^. Aberrational inflammatory processes such as long-lasting inflammation cause abnormal neural connectivity and hyperexcitability that result in the induction of epileptic seizures^5^^,^^6^. Additionally, about one third of the patients are resistant to anti-epileptic drugs that act by different mechanisms. There is an urgent need for new drugs that ensure the complete recovery.

Serotonergic transmission of the brain plays a key role in the regulation of cortical excitatory and inhibitory balance^7^. Data from preclinical and clinical studies indicate that while decreased serotonergic neurotransmission in the brain is implicated in the generation and aggravation of epileptic seizures, the elevated concentrations of synaptic serotonin might exhibit anticonvulsant actions^7^^-^^10^. It was demonstrated that serotonin depletion enhanced spontaneous recurrent seizures and destroyed hippocampal neurons in kainic acid-induced epilepsy rat model^11^. Reduced concentrations of hippocampal serotonin were found in patients with refractory unilateral temporal lobe epilepsy with hippocampal sclerosis who underwent epilepsy surgery^12^. Moreover, epilepsy occurs considerably more frequent among depressed patients compared to the general population^9^. Such serotonergic involvements in the pathophysiology of epilepsy suggest that prominent neuropsychiatric comorbidities of epilepsy such as depression, bipolar disorders, and anxiety can enhance the risk of seizures or vice versa^13^. 

The anticonvulsant effects of enhanced serotonergic tone in the brain are well known. However, mechanisms mediating the action of serotonin and its potential relationship with neuroinflammatory processes remain unclear. We explored the effects and potential mechanisms of action of the modulation of the serotonergic system by serotonin, selective serotonin reuptake inhibitor (SSRI) fluoxetine, and 5-HT1B/D receptor agonist sumatriptan on the neuroinflammatory markers and epileptic seizures in the pentylenetetrazole-induced seizure model in rats. 

## METHODS

### Animals

Ethical approval for animal experiments was obtained from Bolu Abant Izzet Baysal University Animal Experiments Local-Ethics Committee. Male Wistar rats 9-11 weeks old and weighing 190 to 220 g were used in the experiments. Rats were supplied from the Animal House of Bolu Abant Izzet Baysal University, Turkey. Rats were handled in compliance with the Guide for the Care and Use of Laboratory Animals. All rats had free access to a standard rodent feed and water, and were exposed a 12-h light/dark cycle at 22 ± 2°C. 

### Drugs and reagents

Pentylenetetrazole (PTZ), serotonin hydrochloride (5-HT), sumatriptan succinate, fluoxetine hydrochloride, cOmplete™ protease inhibitor cocktail, and phosphate-buffered saline were purchased from Sigma-Aldrich (Schnelldorf, Germany); IL-1β, IL-6, and TNF-α ELISA kits were purchased from ELABscience (Wuhan, P.R. China). Pentylenetetrazole, serotonin, sumatriptan, and fluoxetine were dissolved in physiological saline (0.9% NaCl). 

### Experimental groups, drug administrations and induction of epileptic seizures

**Figure 1.**
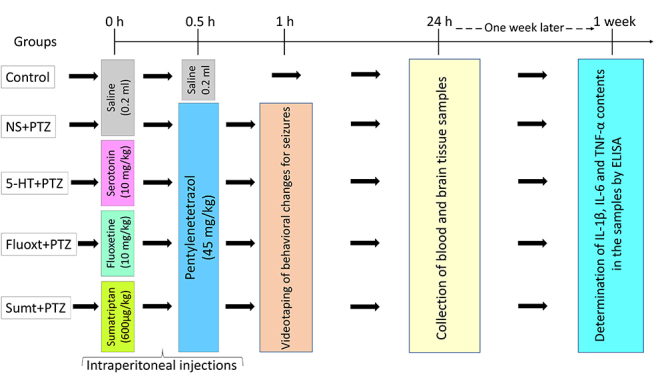
Experimental design and applications.

Rats were randomly divided into 5 groups, as follows: Control (n=6), NS (normal saline)+PTZ (n=9), 5-HT+PTZ (n=8), Fluoxt+PTZ (n=8), and Sumt+PTZ (n=8). We adjusted the number of rats in the groups to obtain at least 6 surviving animals in each group for biochemical analyses, since early death can occur in the PTZ-induced seizure model we used. All drug administrations were performed via the intraperitoneal route. The control group was administered 0.2 mL of physiological saline both at the start time and after 30 min. The other groups were administered 0.2 mL of physiological saline, 10 mg/kg serotonin^14^, 10 mg/kg selective serotonin reuptake inhibitor fluoxetine^14^, and 600 μg/kg 5-HT1B/D receptor agonist sumatriptan^15^, and also after 30 min, all of them were administered 45 mg/kg of pentylenetetrazole^2^^,^^8^^,^^16^^,^^17^ to induce epileptic seizures. Experimental groups and applications are shown in Figure 1. Following PTZ administration, rats were placed in plexiglass cages (40 cm X 40 cm X 30 cm) and videotaped for 30 min to assess behavioral seizures using the Racine's scale. The stages of seizures were assessed by Racine’s scoring (0-5) as previously described^2^^,^^8^: stage 0, absence of response; stage 1, facial movements with saccade of ears and whiskers; stage 2, myoclonic jerks without rearing; stage 3, clonus of one forelimb; stage 4, rearing with bilateral forelimb clonus; stage 5, generalized tonic-clonic seizures. Based on our previous dose adjustment studies and papers of other groups, we determined the dose of PTZ as 45 mg/kg that induced seizure stages specified in the Racine's scale^2^^,^^8^^,^^16^^,^^17^. 

### Collection of blood and brain samples

Since 6 rats survived 24 hours after PTZ administration in PTZ-treated groups, blood and tissue were collected from 6 rats in each group. Briefly, under ketamine anesthesia (90 mg/kg, i.p.), about 5 mL of blood was collected by a syringe from the right ventricle of rats that survived 24 hours after PTZ injection (number of deaths induced by PTZ in the groups is given in Table 1). Shortly after, the head regions of all animals were perfused transcardially with 150 mL heparinized phosphate-buffered saline (PBS, pH 7.4) to remove blood from the brain tissue. Blood samples were immediately centrifuged at 1000 *g* for 15 min at 4°C. The separated serum samples were stored at -80°C for a week until assayed for cytokines. After transcardial perfusion, the cranium of rats was opened and the whole brain without cerebellum was gently harvested. The brain tissue samples were homogenized at 4°C in fixed volumes (100 mg wet tissue/1 mL) of PBS (pH 7.4) containing the protease inhibitor cocktail using a light-duty Ultra-Turrax homogenizer (ISOLAB, Wertheim, Germany). The homogenates were centrifuged at 10,000 g for 15 min at 4°C, and the separated supernatant samples were stored at -80°C until assayed for cytokines.

**Table 1.** The number and percentage of animals with generalized tonic-clonic seizures and deaths induced by PTZ in the groups.

Groups	GTCS		Deaths		Total number of subject
	number	%	number	%	
NS+PTZ	9	100	3*	33.3	9
5-HT+PTZ	8	100	2*	25	8
Fluoxt+PTZ	8	100	2*	25	8
Sumt+PTZ	8	100	2*	25	8

*P=0.973 (Pearson Chi-Square). NS: normal saline; 5-HT: serotonin; fluoxt: fluoxetine; sumt: sumatriptan; PTZ: pentylenetetrazole.

### Determination of IL-1β, IL-6, and TNF-α in brain homogenates by ELISA

The concentrations of IL-1β, IL-6 and TNF-α in brain tissue were determined by enzyme-linked immunosorbent assay kits. The detection limit was ~19 pg/mL for IL-1β, ~38 pg/mL for IL-6 and ~47 pg/mL for TNF-α. The assay was performed in compliance with the instructions of manufacturer and in duplicates. After, 100 μL of sample or IL-1β, IL-6, and TNF-α standard was added to wells of a 96-well plate. The plate was incubated for 1.5 h at 37°C. Next, the liquids in the plate were removed, and instantly 100 μL biotinylated detection Ab solution was added to the wells, and the plate was incubated for 1 h at 37°C. Next, the plate was washed 3 times with the wash buffer, 100 μL HRP conjugate was added to the wells, and the plate was incubated for 0.5 h at 37°C. Next, the plate was washed 5 times with the wash buffer, 90 μL substrate solution was added to the wells, and the plate was incubated for 15 min at 37°C. Then, 50 μL of stop solution was added to the wells. The optical density was determined at 450 nm in the microplate reader (Epoch BioTek Instruments, Inc. Highland Park). 

### Statistical analysis

Data are given as mean±SEM. SPSS statistical package program was used for the statistical analysis of the data (IBM SPSS Statistics for Windows, Version 22.0, IBM Corp, Armonk, NY, USA). Analysis of data distribution was carried out using the Shapiro-Wilk test. The groups were compared by ANOVA followed by Bonferroni post hoc tes, or by Kruskal-Wallis followed by Dunn’s multiple comparison test. A p<0.05 was considered significant. 

## RESULTS

### The effects of serotonergic drugs on the PTZ-evoked seizures

**Figure 2.**
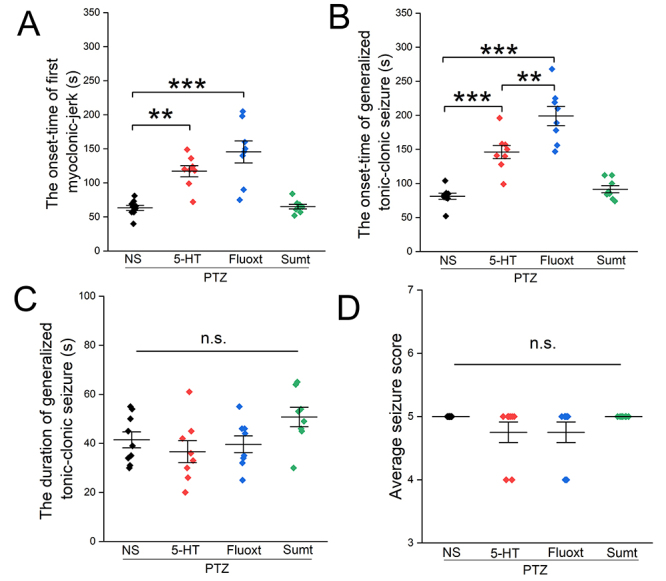
The effects of serotonin, fluoxetine and sumatriptan on behavioral seizures and seizure score in PTZ-induced epileptic rats. **A:** Effects of serotonin, fluoxetine, and sumatriptan on the onset time of first myoclonic jerk; **B:** Onset time of generalized tonic-clonic seizure; **C:** Duration of generalized tonic-clonic seizure; **D:** Average seizure score.

All rats developed seizures following PTZ injection (Table 2). Pre-administration of both serotonin and fluoxetine significantly extended the onset time of the first myoclonic jerk compared with the NS+PTZ group (P=0.001 and P= 0.0001, Figure 2A). However, sumatriptan pre-treatment had no effect on the onset time of the first myoclonic jerk compared with the NS+PTZ group (P=1.0, Figure 2A). 

**Table 2.** Number of rats displaying seizures after PTZ injection.

Seizure stage	Groups			
	NS+PTZ (n=9)	5-HT+PTZ (n=8)	Fluoxt+PTZ (n=8)	Sumt+PTZ (n=8)
Stage 0	0	0	0	0
Stage 1	0	0	0	0
Stage 2	0	0	0	0
Stage 3	0	0	0	0
Stage 4	0	2	2	0
Stage 5	9	6	6	8

NS: normal saline; 5-HT: serotonin; fluoxt: fluoxetine; sumt: sumatriptan; PTZ: pentylenetetrazole; GTCS: generalized tonic-clonic seizures.

**P < 0.01 and ***P < 0.001. ANOVA followed by Bonferroni post-hoc test. NS: normal saline; 5-HT: serotonin; fluoxt: fluoxetine; sumt: sumatriptan; PTZ: pentylenetetrazole; n.s.: non-significance.

In addition, pre-administration of both serotonin and fluoxetine significantly extended the onset time of generalized tonic-clonic seizures compared with the NS+PTZ group (P=0.0001, Figure 2B). Moreover, the effect of fluoxetine was greater than that of serotonin (Fluoxt+PTZ group versus 5-HT+PTZ group, P=0.002, Figure 2B). Sumatriptan pre-treatment did not affect the onset time of generalized tonic-clonic seizures compared with the NS+PTZ group (P=1, Figure 2B).

The pre-administration of serotonin, fluoxetine and sumatriptan had no effect on the duration of generalized tonic-clonic seizures compared with the NS+PTZ group (P=0.08, Figure 2C). Similarly, these drugs did not affect the average seizure score (P=0.196, Figure 2D). Additionally, pre-administrations of serotonin, fluoxetine, and sumatriptan did not affect survival in PTZ-induced epileptic rats compared with the NS+PTZ group (P=0.973, Pearson Chi-Square test, Table 1). Analysis of behavioral parameters of seizures, except for survival rates, was performed by ANOVA followed by Bonferroni post-hoc test. Mean values of drug effects on the behavioral seizures’ parameters are given in Table 3.

**Table 3.** Mean values for effects of the drugs on behavioral seizure parameters.

Groups	Behavioral seizure parameters			
	Onset time of first myoclonic jerk	onset time of generalized tonic-clonic seizure	duration of generalized tonic-clonic seizure	average seizure score
NS+PTZ	63.3±3.8	81.2±4.4	41.4±3.2	5±0.0
5-HT+PTZ	117.1±8.2	146.1±9.8	36.6±4.5	4.7±0.1
Fluoxt+PTZ	145.5±16.1	199.0±14	39.6±3.4	4.7±0.1
Sumt+PTZ	65.1±3.3	91.5±5.2	50.7±3.9	5±0.0

Values are presented as mean ± SEM. SEM: standard error of the mean; NS: normal saline; 5-HT: serotonin; fluoxt: fluoxetine; sumt: sumatriptan; PTZ: pentylenetetrazole; GTCS: generalized tonic-clonic seizures.

### Effects of serotonergic drugs on the concentrations of pro-inflammatory cytokines in the serum and brain tissue in PTZ-induced epileptic rats

**Figure 3.**
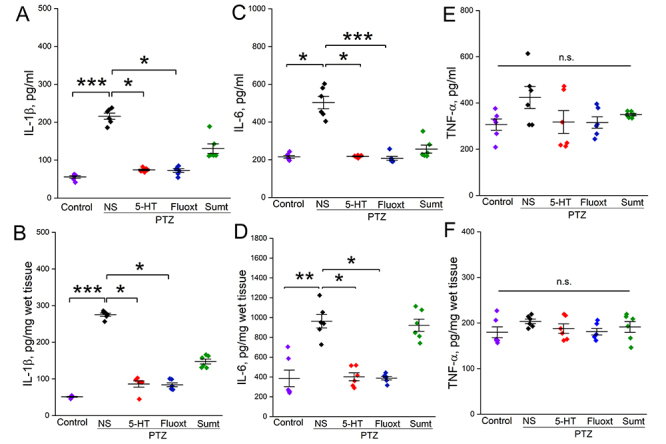
Effects of serotonin, fluoxetine, and sumatriptan on concentrations of pro-inflammatory cytokines in serum and brain tissue in PTZ-induced epileptic rats. Effects of serotonin, fluoxetine, and sumatriptan on concentrations of IL-1β in serum (**A**) and brain tissue (**B**), on the concentrations of IL-6 in serum (**C**) and brain tissue (**D**), on the concentrations of TNF-α in serum (**E**) and brain tissue (**F**). N=6 for each group.

PTZ treatment significantly increased both IL-1β and IL-6 concentrations in both serum (P=0.0001 for IL-1β, Figure 3A and P= 0.014 for IL-6, Figure 3C) and brain tissue (P=0.0001 for IL-1β, Figure 3B and P=0.006, Figure 3D) (NS+PTZ group versus Control group). Contrarily, it did not change TNF-α concentrations both in serum (P=0.145, Figure 3E) and brain tissue (P=0.897, Figure 3F). 

*P < 0.05, **P < 0.01, and ***P < 0.001. Kruskal-Wallis followed by Dunn’s test. NS: normal saline; 5-HT: serotonin; fluoxt: fluoxetine; sumt: sumatriptan; PTZ: pentylenetetrazole; n.s.: non-significance.

On the other hand, pretreatment of both serotonin and fluoxetine significantly reduced the increases in concentrations of both IL-1β and IL-6 induced by PTZ in serum (P=0.033 for IL-1β, Figure 3A and P=0.039 for IL-6, Figure 3C) and brain tissue (P= 0.025 for IL-1β, Figure 3B and P= 0.048 for IL-6, Figure 3D), compared to NS+PTZ, respectively. However, sumatriptan pretreatment did not change IL-1β and IL-6 levels in serum (P=1.0 for IL-1β and IL-6, Figure 3A and C) and brain tissue (P=1.0 for IL-1β and IL-6, Figure 3B and D).

Similar to PTZ treatment, pre-treatments with serotonin, fluoxetine and sumatriptan did not affect the concentrations of TNF-α in serum and brain tissue, (P>0.05 for all comparisons, Figure 3E and F). Data from pro-inflammatory cytokines were analyzed by Kruskal-Wallis followed by Dunn’s multiple comparison test. Mean values for drug effects on the pro-inflammatory cytokines’ parameters are given in Table 4. 

**Table 4.** Mean values for effects of the drugs on biochemical parameters.

Groups	Biochemical parameters					
	IL-1 beta		IL-6		TNF alpha	
	Plasma (pg/mL)	Brain (pg/mg wet tissue)	Plasma (pg/mL)	Brain (pg/mg wet tissue)	Plasma (pg/mL)	Brain (pg/mg wet tissue)
Control	55.5±3.3	51.3±1.2	215.2±7.0	385.7±83.7	306.4±24.3	179.6±12.0
NS+PTZ	215.8±8.2	275.2±4.3	503.2±33.2	963.1±68.2	423.9±47.7	203.6±5.0
5-HT+PTZ	74.2±2.0	86.3±8.5	217.7±2.3	402.3±40.3	317.7±49.3	187.9±10.2
Fluoxt+PTZ	72.3±4.7	84.0±5.2	207.3±10.2	387.8±17.5	315.5±24.6	180.9±7.1
Sumt+PTZ	130.2±12.6	147.4±6.6	256.4±21.0	922.3±61.0	349.6±5.3	191.4±11.8

Values are presented as mean ± SEM. SEM: standard error of the mean; IL: interleukin; NS: normal saline; 5-HT: serotonin; fluoxt: fluoxetine; sumt: sumatriptan; PTZ: pentylenetetrazole; GTCS: generalized tonic-clonic seizures.

## DISCUSSION

In the current study, we showed that both serotonin and fluoxetine, but not sumatriptan, alleviated PTZ-induced seizures by prolonging the onset-times of myoclonic jerks and generalized tonic-clonic seizures, respectively. In addition, the effect of fluoxetine on the generalized tonic-clonic seizures was greater than that of serotonin. On the other hand, PTZ elevated the levels of pro-inflammatory cytokines IL-1β and IL-6, without changing TNF-α levels, in serum and brain tissue. However, pre-treatments with serotonin and fluoxetine, but not sumatriptan, reduced the increases in the levels of IL-1β and IL-6 in serum and brain tissue evoked by PTZ. 

Accumulating preclinical and few clinical evidence indicates that extracellular serotonin enhancement by 5-hydroxytryptophan and SSRIs exhibits anticonvulsant effects^10^. Fluoxetine diminished the spontaneous seizure rate in pilocarpine-induced epilepsy rat model^18^ and reversed status epilepticus-evoked augmentation in brain excitability in lithium-pilocarpine-induced rat model of epilepsy^19^. Direct intrahippocampal administration of exogenous serotonin also suppressed seizures in pilocarpine‐induced seizure model^20^. Additionally, a clinical study reported that frequency of seizures was decreased in patients with epilepsy who received fluoxetine or citalopram^21^. 

Our findings regarding the anticonvulsant effects of serotonin and fluoxetine on the seizures are in line with those previous studies. However, the vast majority of previous studies focused on the effects of endogenous serotonin enhanced by SSRIs rather than exogenous serotonin. Herein, we further confirmed the anticonvulsant action of exogenous serotonin treatment by demonstrating that it extended the onset-times of myoclonic jerks and generalized tonic-clonic seizures in PTZ-induced seizure model. In addition, we found that the anticonvulsant effect of endogenous serotonin enhanced by fluoxetine on generalized tonic-clonic seizures was greater than that of exogenous serotonin. This may be due to the fact that fluoxetine may elevate the extracellular serotonin concentration in the brain more than exogenous administration of serotonin. Additionally, the intraperitoneal application of serotonin may preclude the substance to reach the brain due to enzymatic degradation.

Increasing evidence suggests that the anti-seizure actions of serotonin are mostly mediated by 5-HT1A, 5-HT2C, and 5-HT3 receptors^10^^,^^22^^,^^23^. For instance, a preclinical study demonstrated that two 5HT1A receptor agonists, 8-OH-DPAT and indorenate, exhibited therapeutic effects against epileptic activity depending on the type of seizure in three different animal models of epileptic seizures^23^. However, activation of 5-HT1B/D receptors were reported to exert dual effects on seizures^24^^,^^25^. 5-HT1B/D receptor agonist sumatriptan exhibited biphasic effect on seizures in a dose-dependent manner in PTZ-evoked seizure model in mice^25^. In the current study, although we chose the most probable dosage for potential anticonvulsant effect^15^^,^^25^, activation of 5-HT1B/D receptors by sumatriptan did not exert pro-convulsant or anti-convulsant action. Thus, more studies are needed to clarify these dilemmas.

On the other hand, it is well established that neuroinflammation, including enhanced release of pro-inflammatory cytokines such as IL-1β, IL-6 and TNF-α, plays a central role in epileptogenesis^3^^-^^6^. It was stated that IL-1β increases neuronal over-excitability by promoting glutamate release from astrocytes, and by decreasing its re-uptake^26^. It was shown that intranasal treatment of IL-6 exerted pro-convulsive impacts in PTZ-induced seizure model^27^. Additionally, TNF-α increased the expression of AMPA receptors and triggered GABA receptor endocytosis in hippocampal neuronal cultures^28^, thus facilitating seizure generation by enhancing the excitatory tone in the brain. Moreover, it was found that lasting febrile seizures elevated cerebrospinal fluid levels of IL-1β, IL-6, and TNF-α in patients^29^. Enhanced concentrations of IL-1β in epileptogenic brain tissue specimens of patients with temporal lobe epilepsy lowered GABA-mediated neurotransmission and stimulated the initiation of seizures^30^. 

Studies on whether the serotonergic system modulation exerts its anticonvulsant effects through neuroinflammatory processes are very limited. We found that serotonin and fluoxetine, but not sumatriptan, decreased PTZ-induced increases in IL-1β and IL-6 levels in serum and brain tissue. In addition, although not statistically significant, 5-HT and fluoxetine decreased seizure severity. Therefore, we can speculate that depending on the decrease in seizure severity, IL-1β and IL-6 levels may have decreased in both 5-HT and fluoxetine groups. Furthermore, the seizure severity was stage 5 in the sumatriptan-pretreated group and sumatriptan did not decrease the levels of these pro-inflammatory cytokines. Hence, these findings also clearly support our speculation. Our findings are in line with the previous studies mentioned above. Moreover, this anti-neuroinflammatory effect of serotonin further strengthens our findings regarding its anti-seizure effects and opens new insights about potential mechanisms of action of serotonergic modulation on the epileptic seizures.

Serotonin is a high affinity natural ligand for 5-HT receptors including 5-HT1B/D receptors. In addition to their neuropsychiatric effects such as major depressive disorder and anxiety states, activation of 5-HT1B/D receptors mediates anti-neuroinflammatory effects mainly through inhibition of vasoactive neuropeptide calcitonin and gene-related peptide release from trigeminal sensory fibers^31^^,^^32^^,^^33^. On the contrary, in the present study, activation of 5-HT1B/D receptors by sumatriptan did not display anti-neuroinflammatory effects. This may be due to the lower expression of 5-HT1B/D receptors in neurons in the region of seizure generation. However, endogenous or exogenous serotonin exhibited anti-neuroinflammatory effects, suggesting that this effect may be a common consequence of activation of multiple 5-HT receptors by serotonin.

Our findings regarding anti-inflammatory effects of serotonin and fluoxetine are in accordance with previous studies reporting their potential immunoregulatory functions. It was reported that serotonin represses the release of TNF-α and IL-1β through 5-HT receptors in monocytes/macrophages^34^. Moreover, it was demonstrated that serotonin inhibited lipopolysaccharide-stimulated pro-inflammatory cytokine production from human macrophages by 5-HT7-PKA axis^35^.

SSRI sertraline reduced PTZ-induced increases in IL-1β and TNF-α mRNA expressions in rat hippocampus^36^. In contrast, in our study, PTZ did not lead to an increase in TNF-α levels in serum or brain tissue. Likewise, the other administrations also showed no effect. We measured the TNF-α peptide, but not mRNA. Therefore, the reason for this difference may be that TNF-α peptide may degrade faster than its mRNA. On the other hand, sumatriptan showed no effect on PTZ-induced increases in IL-1β and IL-6 levels as well as on PTZ-induced behavioral seizures. 

Compared to the PTZ group, these unchanged IL-6 and IL-1β levels in the sumatriptan plus PTZ group may be due to the seizure effect, since seizure severity did not decrease in this group. Moreover, we measured these pro-inflammatory cytokines in plasma and brain tissue after PTZ injections following pretreatment of serotonergic drugs rather than shortly before PTZ injection. Therefore, PTZ administered after sumatriptan pretreatment may have suppressed the effect of sumatriptan. However, our current findings suggest that anticonvulsant and anti-neuroinflammatory effect of serotonin may not be mediated by 5-HT1B/D receptors. 

Taken together, our findings are of great importance in terms of treatment of epilepsy and its neuropsychiatric comorbidities, as epilepsy patients experiencing long-lasting active epilepsy have higher rates of neuropsychiatric comorbidities such as depression, bipolar disorders, and anxiety^13^. Drugs targeting monoamines such as serotonin are commonly used in the treatment of these neuropsychiatric comorbidities. Therefore, drugs targeted at increasing serotonin levels in the synaptic cleft, such as SSRIs, may be useful for treatment of both epilepsy and its neuropsychiatric comorbidities by acting as anticonvulsant and antidepressant. 

In conclusion, our findings suggest that endogenous and exogenous serotonin exhibit anticonvulsant effects by suppressing neuroinflammation in PTZ-induced seizure model. It seems that 5-HT1B/D receptors are not responsible for these effects of serotonin.
